# Filarial Parasites Develop Faster and Reproduce Earlier in Response to Host Immune Effectors That Determine Filarial Life Expectancy

**DOI:** 10.1371/journal.pbio.1000525

**Published:** 2010-10-19

**Authors:** Simon A. Babayan, Andrew F. Read, Rachel A. Lawrence, Odile Bain, Judith E. Allen

**Affiliations:** 1 Centre for Immunity, Infection and Evolution, University of Edinburgh, Edinburgh, United Kingdom; 2 Institute of Immunology and Infection Research, School of Biological Sciences, University of Edinburgh, Edinburgh, United Kingdom; 3 Center for Infectious Disease Dynamics, Departments of Biology and Entomology, The Pennsylvania State University, University Park, Pennsylvania, United States of America; 4 Fogarty International Center, National Institutes of Health, Bethesda, Maryland, United States of America; 5 Royal Veterinary College, University of London, London, United Kingdom; 6 USM 307, Parasitologie comparée et Modèles expérimentaux, Muséum National d'Histoire Naturelle, Paris, France; Stanford University, United States of America

## Abstract

Humans and other mammals mount vigorous immune assaults against helminth parasites, yet there are intriguing reports that the immune response can enhance rather than impair parasite development. It has been hypothesized that helminths, like many free-living organisms, should optimize their development and reproduction in response to cues predicting future life expectancy. However, immune-dependant development by helminth parasites has so far eluded such evolutionary explanation. By manipulating various arms of the immune response of experimental hosts, we show that filarial nematodes, the parasites responsible for debilitating diseases in humans like river blindness and elephantiasis, accelerate their development in response to the IL-5 driven eosinophilia they encounter when infecting a host. Consequently they produce microfilariae, their transmission stages, earlier and in greater numbers. Eosinophilia is a primary host determinant of filarial life expectancy, operating both at larval and at late adult stages in anatomically and temporally separate locations, and is implicated in vaccine-mediated protection. Filarial nematodes are therefore able to adjust their reproductive schedules in response to an environmental predictor of their probability of survival, as proposed by evolutionary theory, thereby mitigating the effects of the immune attack to which helminths are most susceptible. Enhancing protective immunity against filarial nematodes, for example through vaccination, may be less effective at reducing transmission than would be expected and may, at worst, lead to increased transmission and, hence, pathology.

## Abstract 2

During larval development, filarial nematodes adjust their lifelong reproductive strategy to the presence of anti-parasitic immune cells that determine host resistance and experimental vaccine efficacy.

## Author Summary

Many organisms are able to adapt their development to the severity of their environment based on specific cues, and we have identified such a phenomenon, termed phenotypic plasticity, in the filarial parasite *Litomosoides sigmodontis*. Filarial nematodes infect about 200 million people worldwide, and much effort is going into finding a vaccine that would complement current drug treatments. Although anti-filarial immunity can be achieved, we show, in accord with evolutionary theory, that when these parasites infect a new host, they are able to adjust their development and reproduction to the presence of immune cells specialized in anti-helminth attack. These developmental schedules are determined within hours and impact their lifelong reproductive strategy; when immune attack is strong, and thus mortality is likely to be high, they produce offspring earlier and in greater numbers. Because current experimental vaccines rely on the very immune elements to which these nematodes adjust their development, their phenotypic plasticity could mitigate the expected reduction of disease burden in vaccinated populations.

## Introduction

Facultative alterations in reproductive and developmental schedules are an important mechanism by which animals optimize their lifetime reproductive output in the face of environmental heterogeneities that determine mortality [1],[2]. For instance, in the presence of predatory fish, *Daphnia* (small freshwater crustacean) adjust their age and size at maturity to maximize reproductive output for a given local predation risk [3],[4]. Similarly, *Nucella lamellosa* marine snails only display their full defensive phenotype when they detect the soluble products of both predatory crabs and the debris of conspecific snails [5]. The evolution of adaptive phenotypic plasticity of this kind requires fitness-relevant environmental heterogeneity, detectable environmental cues that reliably predict future survival, and the existence of life history strategies that mitigate the consequences of altered life expectancy [6]–[11]. Evolutionary biologists have suggested that all three requirements will be met in parasitic helminths [12]–[16].

Despite the renowned ability of helminths to modulate the immune responses of their host [17],[18], the amplitude and profile of the immune response remain largely predictive of parasite mortality [19]–[22]. The strength of protective responses mounted by hosts against parasitic attack will depend on host factors such as level of prior exposure, age, sex, and condition [23], with the consequence that parasite life expectancy can vary substantially among hosts. Thus, helminths can encounter hosts in which they will have either long or short life spans. There are a variety of reports that helminth development is enhanced by host immune molecules [14],[24]–[33], raising the possibility that invading helminths could be adjusting their developmental and reproductive schedules in order to minimize the fitness consequences of impending immune attack. All else being equal, the expectation is that parasitic helminths should reproduce earlier in hosts where life-threatening responses are already present [8],[13],[14]. However, this has yet to be tested in a suitable experimental setting, despite its relevance for disease control. For instance, alterations in reproductive schedules in immunized hosts may reduce and possibly even negate the impact of non-sterilizing vaccines on disease transmission [12],[34].

Filarial nematodes can cause debilitating diseases in humans such as river blindness and elephantiasis [35],[36]. Their successful establishment, survival to sexual maturity, and reproduction are determined by the host's adaptive immune response, which they evade, modulate, and suppress [17],[18],[37]. Host immune responses to helminth infections are complex, but IL-5 driven polynuclear eosinophils are a primary effector cell type thought to be responsible for parasite death [20]–[22],[32],[33],[38]–[43]. In *Litomosoides sigmodontis* infections (see life cycle in Figure S1), IL-5 driven eosinophils are responsible for the vaccine-mediated killing of the larvae at the outset of infection in conjunction with adaptive immune responses [33],[44],[45] and for negatively impacting the survival of adults at sexual maturity in both immunized and non-immune hosts [39]. Mice that constitutively overexpress IL-5 have an increased eosinophilia that causes a more rapid clearance of *L. sigmodontis* [32]. Conversely, in mice lacking IL-5 and/or functional eosinophils, the parasites survive and reproduce well beyond their normal life span in control hosts [39],[40]. However, we have previously found that eosinophil-rich inflammation at the cutaneous site of inoculation, such as that induced by vaccination, rarely induces full protection and, counterintuitively, that this inflammation triggers faster larval development of the parasites that do survive [31],[44], as assessed by their length and stage. Enhanced development (which hereafter refers to both growth and moulting) in the presence of strong eosinophil-rich immune responses was contrary to our initial expectations but is consistent with evolutionary predictions [12]–[14] that nematodes will respond to environmental cues predictive of an enhanced risk from immune attack and, consequently, will alter their reproduction in order to maximize offspring production before immune clearance.

In this study we determine that variations in filarial larval size and stage are a plastic response to an early, local, and transient predictor of their host's immune response; we determine that eosinophils, which are necessary for immune clearance of filarial infections, act as a developmental cue and that adaptive immunity, IL-5, and IL-4 contribute to accelerating early parasite growth despite their role in stunting later development; and finally we show how those early variations in the nematodes' life history traits alter their fecundity.

## Results/Discussion

### Filarial Nematodes Develop Faster When IL-5 and Eosinophils Are Present

**Figure 1:**
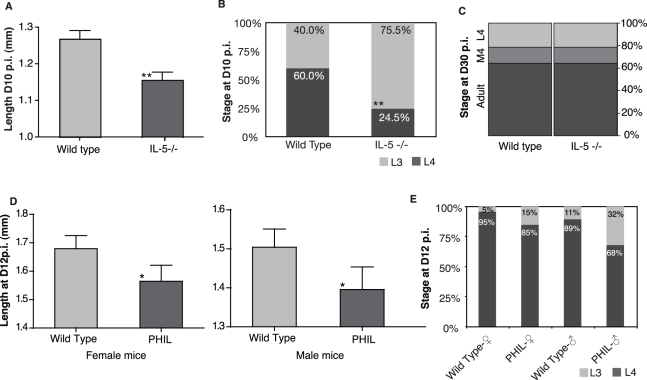
Filarial nematodes developed faster when IL-5 driven eosinophils were present. *Litomosoides sigmodontis* filarial nematodes developed slower during their larval stages in IL-5 deficient (IL-5^−/−^) mice than in C57BL/6 wild type controls as measured (A) by their shorter lengths (** *p* = 0.015, ANOVA; *n* = 50 larvae nested in 5 mice per group) and (B) by their delayed moulting to the 4^th^ larval stage at D10 p.i. (** *p* = 0.0007, Chi^2^ test; *n* = 5 mice). (C) At D30 p.i., however, no differences in the moulting rate to the adult stage were observed between IL-5^−^/^−^ mice and wild type controls (*n* = 5 mice). The constitutive absence of eosinophils in PHIL mice resulted in slower larval development as judged by (D) their lengths in both male and female mice (*, *p* = 0.04 for the effect of mouse strain when variation due to mouse sex is accounted for, GLM; *n* = 57 to 59 in 7 mice) and by (E) their moulting rates (*p* = 0.02, Fisher Exact Test; *n* = 7 mice) at D12 p.i. as compared to C57BL/6 wild type controls. None of the treatments affected larval survival (see Figure S2A and S2B). Error bars depict s.e.m.

Interleukin-5 (IL-5), a major element of the T helper 2 (Th2) type effector response, is responsible for vaccine-induced protection and resolution of filarial infection [22],[33],[39] and thus a likely candidate for the developmental cue used by *L. sigmodontis* [33],[44]. In homozygous IL-5 deficient mice (IL-5^−/−^), the absence of IL-5 had no effect on the establishment of the filariae when compared to C57BL/6 wild type controls, confirming previous data in primary infections (Figure S2A) [45]. However, 10 d post infection (D10 p.i.), filarial development was delayed in the IL-5 deficient mice, as larvae were significantly smaller (Figure 1A), and fewer had reached the fourth larval stage (L4) (Figure 1B) than in wild type controls. However, at D30 p.i., the proportions of the different stages were identical (20% L4, 15% undergoing their moult, and 65% adults; Figure 1C), suggesting that early growth retardation is not necessarily permanent. Because IL-5 acts through eosinophils to kill filarial parasites, these cells may mediate the early variations in larval development. Furthermore, there have been reports that eosinophilia correlates with the size of another nematode, *Teladorsagia circumcincta* [28]. To confirm that IL-5 was acting via eosinophils, we inoculated *L. sigmodontis* into PHIL mice that lack the eosinophil lineage entirely [46]. In these mice the filariae developed slower than in wild type C57BL/6 controls as measured by both their lengths and moulting rate (Figure 1D and 1E). Given our previous findings that no difference in larval development is observed between large and small doses of infective larvae [47], resource availability is unlikely to explain the observed differences. Taken together, these results show that the growth and moulting acceleration mediated by IL-5 and eosinophils are morphologically detectable in the early phases of larval development only, and that variations in larval development are not due to differential survival nor to competition for resources between the infective larvae.

### Larval Development Schedule Was Determined by IL-5 Dependant Eosinophilia at the Earliest Encounter with the Definitive Host

**Figure 2:**
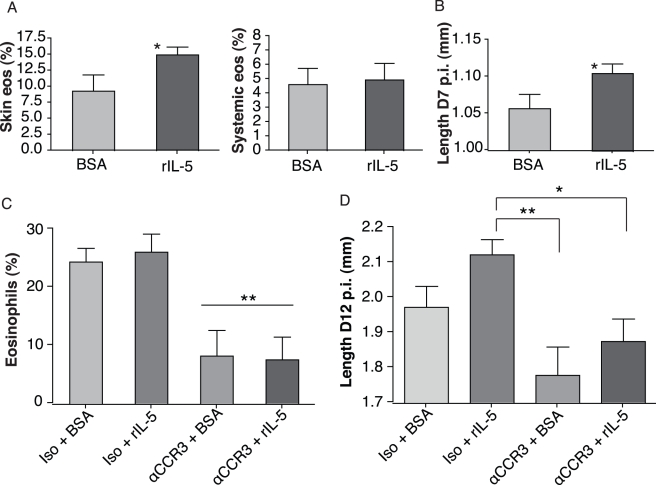
Filarial nematodes responded to the presence of IL-5 driven eosinophils at the outset of infection. (A) Topical injection of recombinant IL-5 (rIL5) resulted in a local subcutaneous increase (*p* = 0.05, Wilcoxon rank-sum test, *n* = 5) but no systemic increase in eosinophil recruitment relative to other lymphocyte populations. (B) The addition of rIL5 upon inoculation of infective larvae to BALB/c mice accelerated their growth before their 3rd larval moult, at D7 p.i. (* *p* = 0.019, unpaired two-tailed *t*-test; *n* = 30, no significant effect of mouse). This occurred independently of mouse genetic background as similar data were obtained in BALB/c and in C57BL/6 mice. (C) The depletion of eosinophils by α-CCR3 antibody treatment 24 h before infection resulted in a prolonged reduction of eosinophilia and (D) in a slower larval development that were not rescued by the addition of rIL-5 (*p* = 0.003, ANOVA and Dunn's multiple comparison post test: ** *p*<0.01; * *p*<0.05; *n* = 19 to 23). None of the treatments affected larval survival (see Figure S2C). Error bars depict s.e.m.

We then wanted to establish how soon *L. sigmodontis* life-history traits were determined by their new environment because larvae migrate away from the inflamed subcutaneous tissue within hours of their inoculation [48], reaching the pleural cavity within 4 d while still at the L3 stage [31]. Our results above implicate IL-5 driven eosinophils in accelerating the parasites' development, either directly or through their downstream products. We thus included recombinant IL-5 (rIL-5) in the inoculum containing infective larvae. This would ensure that the parasites be exposed to rIL-5 only until they migrated away or until rIL-5 was degraded—thus for no more than 4 d. We confirmed that the administration of rIL-5 increased local subcutaneous eosinophilia in comparison to a standard protein control of bovine albumin (BSA), while no systemic increase in eosinophilia was observed (Figure 2A). Systemic concentrations of IL-4, IL-5, IL-10, IFN-γ, IgG1, and IgG2a were unaffected by the administration of rIL-5 (unpublished data). This transient presence of rIL-5 and eosinophils resulted in accelerated growth of the larvae as early as D7 p.i. when compared to BSA controls in BALB/c mice (Figure 2B) and in C57BL/6 mice (unpublished data). Consequently, filarial nematodes are able to adjust their development to the immune environment as soon as they enter the host, and this effect is independent of mouse genetic background.

Since IL-5 stimulates the production and recruitment of eosinophils [49], which in turn produce IL5 themselves, we wanted to assess whether eosinophils were solely responsible for our observation that the local addition of rIL-5 correlates with faster filarial developmental. Twenty-four hours before inoculation we selectively depleted eosinophils in BALB/c mice with CCR3-specific monoclonal antibodies that have been shown to deplete no other cell type [50]. Eosinophil recruitment was abolished and remained strongly impaired during the first 12 d of infection (Figure 2C), while neither IL-5 concentrations nor those of IL-4, IL-10, or IFN-γ were significantly affected (unpublished data). Parasite establishment was altered by neither anti-CCR3 nor rIL-5 treatment (Figure S2C), as expected from previous work [33],[45], but larvae inoculated into anti-CCR3-treated mice grew slower than in mice treated with relevant controls (Figure 2D). The addition of rIL-5 accelerated the larvae's growth in mice with intact eosinophils but failed to restore fast developmental rates in anti-CCR3-treated animals (Figure 2D). Indeed, in all anti-CCR3-treated animals, parasites were much smaller than in control animals.

These results suggest that eosinophils, rather than IL-5, provide the developmental cue that *L. sigmodontis* larvae detect in their host and that larvae are capable of responding phenotypically to the presence of eosinophils and/or their products as soon as they enter their host.

### Adaptive Immunity Triggers Faster Larval Development But Is Not Obligatory for Optimal Worm Development

**Figure 3:**
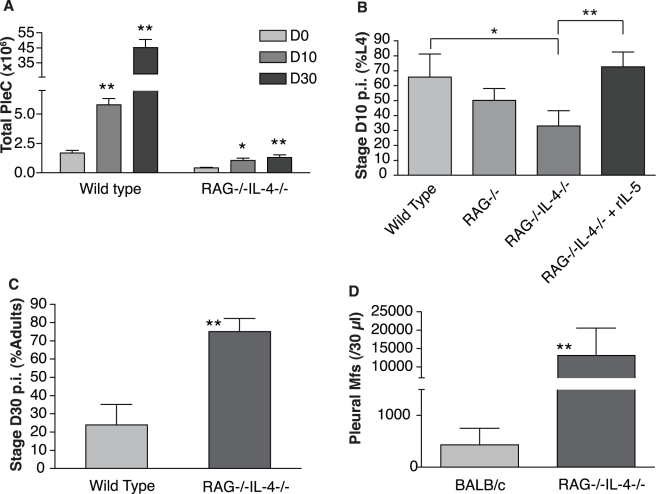
Adaptive immunity accelerates early larval development of *L. sigmodontis* despite impeding its sexual maturation. (A) Cell recruitment increased significantly over time during infection when filariae were inoculated into *rag* ^−/−^, *il4* ^−/−^ and double deficient (*rag* ^−/−^ *il-4* ^−/−^) mice that lack T cells, B cells, and IL-4, but was severely lower overall in *rag* ^−/−^ *il-4* ^−/−^ than in C57BL/6 wild type controls. (B) Filarial larvae in *rag* ^−/−^ *il-4* ^−/−^ mice developed slowest unless rIL-5 was added upon their delivery to the host. (C) However, by D30 p.i. the parasites in *rag* ^−/−^ *il-4* ^−/−^ mice had compensated for their slower development and reached the adult stage earlier than in wild type controls, likely due to the continuous attack by eosinophils and other inflammatory cells in the control mice (see Figure S3A). (D) This resulted in the release of more offspring in *rag* ^−/−^ *il-4* ^−/−^ mice than what is observed in susceptible immunocompetent BALB/c mice. * *p*<0.05; ** *p*<0.01, Wilcoxon rank-sum test; *n* = 5–10 mice per group. Error bars represent s.e.m.

In endemic areas, where individuals are constantly exposed to infective larvae, rarely would filarial nematodes encounter solely innate immune responses. Exposed individuals typically mount adaptive Th2 lymphocyte responses characterized by the production of IL-4, which is needed for Th2 effector function and is a major factor in the production of IL-5 and, thus, in anti-filarial protective immunity [35],[51]. Moreover, in vaccinated mice the adaptive immune system is responsible for killing incoming larvae, both through IL-5-producing Th2 cells and antibody-producing B cells [52]. IL-5 has also been shown to induce B cell maturation and antibody production [53]. Improved development of the filarial nematode *B. malayi* as well as the trematode *Schistosoma mansoni* have been linked to the presence of both B and T cells [27],[29],[30]. It is thus possible that filarial nematodes cue directly into concentrations of IL-4 and/or T and B cells as well as IL-5 or, alternatively, that T and B cells and IL-4 could affect worm developmental schedules through their downstream effects on IL-5 and eosinophils. To specify the role of adaptive immunity in filarial development, and whether IL-5 accelerates larval development only in the presence of T or B cells and/or IL-4, we analyzed larval moulting rates in C57BL/6 *rag* ^−/−^ mice that have neither T nor B cells and *rag* ^−/−^ *il-4* ^−/−^ mice that additionally lack IL-4. These latter mice are therefore almost totally immune deficient in the context of filarial infections. As expected, *rag* ^−/−^ *il-4* ^−/−^ mice failed to recruit leukocytes to the site of infection (Figure 3A), and the eosinophilic response in particular was weaker than in control mice (Figure S3A). At D10 p.i., larval development in *rag* ^−/−^ *il-4* ^−/−^ mice was slower than in wild type mice (Figure 3B), while development in *rag* ^−/−^ mice was intermediate. However, injecting larvae concurrently with rIL-5 restored their developmental rate in *rag* ^−/−^ *il-4* ^−/−^ mice to the levels observed in wild type control mice. No difference in overall parasite survival was observed between groups (Figure S3B). However, long-term exposure to large numbers of leukocytes, and especially eosinophils, is known to stunt filarial nematodes [33],[39],[47],[54]. Indeed, by D30 p.i., the negative effect of the adaptive immune response on parasite development was evident in the wild type C57BL/6 mice, which are non-permissive to patent infection with *L. sigmodontis*. In contrast, the parasites in *rag* ^−/−^ *il-4* ^−/−^ mice had more than compensated for their early slow development (Figure 3C). By D60 p.i. the number of microfilariae in *rag* ^−/−^ *il-4* ^−/−^ mice exceeded that in BALB/c wild type mice by a factor of 30 (Figure 3D), consistently with the known role of adaptive immunity and IL-4 on microfilariae survival [35].

Thus, although adaptive Th2 immunity contributes to accelerating larval development of *L. sigmodontis*, it is not obligatory as IL-5 alone can modify the parasites' developmental schedules. Indeed, in the absence of adaptive immunity, *L. sigmodontis* achieved greater fertility than in the most permissive immunocompetent mouse strain. This provides a confirmation that immune-dependant developmental acceleration is a sign not of better health but of a fitness-enhancing developmental strategy of the parasite. These data are in contrast to what has been observed in *Schistosoma mansoni* infections, in which the host's T cells appear to provide a resource required for normal worm development and transmission in mouse models and in humans [25],[27],[55]. In *rag* ^−/−^ mice, Schistosomes acquire a profoundly abnormal phenotype with reduced body size and reduced fecundity [26],[56] that suggests they have become dependant on the ubiquitous presence of the host's adaptive immune system. *L. sigmodontis*, on the other hand, displays facultative developmental schedules, and we hypothesize that this allows an optimal maturation schedule given the hosts' immune status at the moment of infection.

### Protective Immunity Causes Earlier Onset of Patency and Increased Microfilaraemia

**Figure 4:**
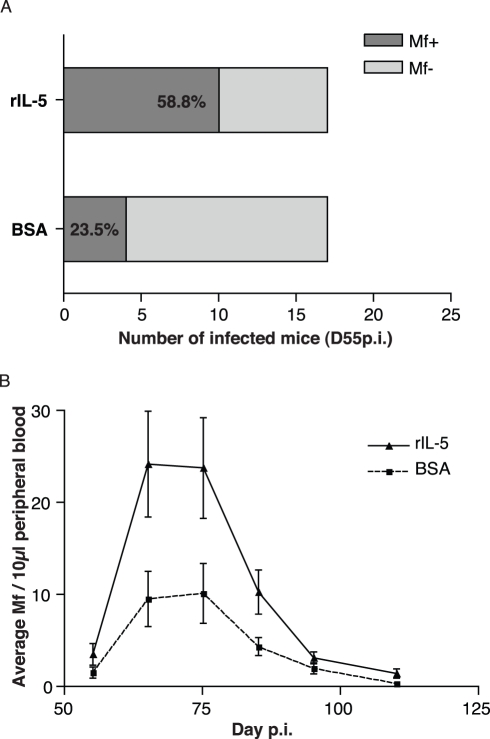
Early eosinophilia enhances *L. sigmodontis* reproductive output. (A) When co-inoculated with eosinophilia-inducing rIL-5 and L3 parasites, BALB/c mice became microfilaraemic sooner than in control infections as suggested by the proportion of mice presenting blood circulating microfilariae by D55 p.i. (*p* = 0.08, Fisher's exact test, *n* = 17, analysis restricted to mice that became microfilaraemic). (B) Early rIL-5-induced eosinophilia resulted in increased microfilaraemia throughout patency (effect of treatment on microfilaraemia *p* = 0.0001, negative binomial glm; *n* = 12, data points represent means ± s.e.m.) and a marginally earlier peak in microfilaraemia (occurring on day 68.5±1 and 72.8±2 in treated mice and controls, respectively, *p* = 0.09).

If larval developmental plasticity in the face of protective immune responses is indeed an adaptive (fitness-enhancing) trait of *L. sigmodontis*, the IL-5 mediated acceleration of parasite development should lead to greater reproduction earlier in infection [12]. We thus assessed the relationship between the presence of IL-5 and eosinophils at the site of inoculation and worm fertility 2 mo later. We injected larvae together with rIL-5 to mice as described above to ensure that eosinophilia would peak locally and early in the infection and then return to levels of control mice thereafter. When larvae were injected with rIL-5, the onset of patency (detection of microfilariae) occurred earlier than in control infections (Figure 4A). After D70 p.i., no difference in microfilaria prevalence was observed between treatment groups. Thus, a local and transiently increased eosinophilia reduces the age at which females are able to release microfilariae into the peripheral circulation. The faster larval development triggered by the addition of rIL-5 upon infection (see Figure 2B, 2D) also resulted in an increased microfilaraemia in the peripheral blood compared to control mice throughout patency (Figure 4B). Additionally, because IL-4 has been shown to specifically control microfilaraemia [39], we wanted to assess whether our observations were due to a rIL-5-driven alteration of IL-4 in susceptible genotypes. In both wild type and IL-4^−/−^ BALB/c mice, rIL-5 treatment increased overall microfilaraemia 5–8-fold over BSA-injected controls (Table 1). While there were vastly superior numbers of circulating microfilariae in IL-4^−/−^ mice as compared to BALB/c mice, in both strains rIL-5 treatment resulted in a similar increase of the overall number of microfilariae in the peripheral circulation throughout patency (Table 1). Thus the impact of early eosinophilia on fecundity was independent of the effector pathways associated with immunity against microfilariae.

**Table 1:** The addition of rIL-5 increased parasite fecundity independently of IL-4.

	BSA	rIL-5
BALB/c	28.5±6.6	66.7±14.1
BALB/c IL-4^−/−^	254.8±83.4	425.6±189.2

BALB/c mice, *n* = 16 per group pooled from three experiments (no significant variation between experiments), and BALB/c *il-4* ^−/−^ mice, *n* = 6 per group, were infected subcutaneously with *L. sigmodontis* larvae that were allowed to develop and reproduce for 150 d. Numbers represent mean cumulative microfilarial loads ± s.e.m. Effects of rIL-5 treatment and of mouse strain were both significant (*p* = 0.009 and *p*<0.0001, respectively), and there was no interaction between treatment and strain (negative binomial glm).

Since IL-5-dependent eosinophilia is a good predictor that hosts are mounting life-shortening immune responses [22],[32],[39],[57]–[59], our data are consistent with the hypothesis [13],[60] that parasitic worms will develop faster in hosts where they can predict that their life spans will be shortened. Our experimental manipulations created a local and transient increase in eosinophilia that had no effect on worm survival but did accelerate their larval development. This is consistent with previously published data showing in primary infections no detectable impact of IL-5 or eosinophils on the survival of infective larvae [32],[45]. Therefore, we did not select larger or fitter larvae (or their odds of surviving would have differed) but induced a developmental reaction to the presence of eosinophils.

However, a corollary of this hypothesis of adaptive phenotypic plasticity is that there must be a trade-off between accelerated larval development and other worm life-history traits [13], otherwise *L. sigmodontis* would always grow faster, irrespective of eosinophilia. Our experiments were designed to investigate developmental rates and fecundity, and therefore can necessarily not examine all aspects of fitness in which this cost may be manifested. It could be that faster larval development itself shortens worm lifespan, perhaps because of direct physiological costs, or insufficient investment in immunosuppression. It may also be that fast larval development reduces offspring viability in subsequent hosts. However, in addition to the limitations of our model system, the costs of phenotypic plasticity are often weak and have rarely been observed in the wild [61]. An alternative hypothesis is that rather than being the result of developmental plasticity, the phenotypic effects we observe are a consequence of *L. sigmodontis* being poorly adapted to our control animals. It could be, for example, that the Th2/IL-5 driven eosinophilic response provides an essential resource for the parasites to develop and reproduce without which they are stunted. This hypothesis fails to explain why *L. sigmodontis* achieves greater reproductive success in mice lacking IL-5 [22],[39], IL-4 (Table 1), or adaptive immune responses (Figure 3) than it does in immunocompetent controls, despite their slower initial larval development (Figure 1A–B, Figure 3B–D). Nonetheless, further study is warranted to definitively distinguish between the adaptive and non-adaptive hypotheses. The adapationist hypothesis is that the worms produce a developmental schedule that maximizes fitness in the immune environment they find themselves; if this hypothesis is correct, our experimental approach essentially “tricks” the worms into undergoing a developmental schedule appropriate to an immune environment more potent than the one they are truly in. Fully evaluating the fitness consequences of eosinophil-triggered developmental plasticity requires an assessment of the phenotypic responses to eosinophils on the longevity, fecundity, and fitness of future generations of faster developing parasites in naïve and immunized hosts.

Our findings may have implications for public health, insofar as blood circulating microfilariae are the transmission stage and a major cause of pathology [35]. We have shown that filarial parasites alter their developmental and reproductive schedules in response to host immune factors in a manner expected to maximize their fitness. Current experimental vaccines rely on the very immune elements that these nematodes use as developmental cues. Unless vaccines can successfully induce sterilizing immunity, facultative life history responses of the sort we have demonstrated here will likely constrain the transmission-blocking that could otherwise be achieved by widespread immunization. In the limit, plastic life history responses could completely negate any expected reduction of the number of secondary infections generated by an initial infection of a vaccinated patient [62]. Rightly, clinical trials analyze the impact of potential vaccines on host health and sometimes antigenic escape of the parasite; we suggest they should also study their effects on parasite life history.

## Materials and Methods

### Mice and Infections

Wild type BALB/c and C57BL/6 mice were bred in house or purchased from Harlan UK. Mice homozygous for disrupted alleles encoding IL-4 (*il4* ^−/−^), IL-5 (*il5* ^−/−^), or RAG2 (*rag* ^−/−^) were bred and housed on site. *il-4* ^−/−^ and *rag* ^−/−^ C57BL/6 mice were crossed and the resulting double knock-outs were maintained on site. PHIL mice on the C57BL/6 background devoid of eosinophils [46] were maintained at the Royal Veterinary College, London. BALB/c mice were used for all experiments involving parasite fitness-relevant assessment, because they are permissive to the sexual maturation and reproduction of *Litomosoides sigmodontis*. BALB/c mice and C57BL/6 mice (wild type and genetically modified) were used for assessing effects on larval development according to mouse availability. Precautions were taken to ensure that those two genetic backgrounds did not confound our developmental observations by repeating key experiments on both strains. All experimental mice were females except for PHIL mice that had equal numbers of each sex in all groups. All mice were kept in individually ventilated cages and age-matched to 6–8 wk old at the time of infection. All experiments complied with the Animals (Scientific Procedures) Act 1986.

### In Vivo Filarial Infections

*Litomosoides sigmodontis* [63] was used for all in vivo experiments (see life cycle in Figure S1). *L. sigmodontis* was maintained in the jird *Meriones unguiculatus* and the mite *Ornithonyssus bacoti* as described previously [64]. Twenty-five to 40 infective L3 were inoculated subcutaneously into laboratory mice, with 4 ng of either recombinant mouse IL-5 (rIL-5) resuspended in PBS-BSA 0.1% or concentration-matched BSA alone to control for protein quantity. Autopsy dates were determined by the *L. sigmodontis* life cycle: the L3 migrates through the lymphatic system to the pleural cavity within 4 d [31] and moults 7–12 d post inoculation (p.i.) and again 28–35 d p.i. into the L4 and adult stages, respectively [64]. Microfilariae become detectable after D50 p.i. *L. sigmodontis* larvae and adults were extracted from the pleural cavity of infected mice and their survival rate was calculated as previously described [31]. Briefly, the pleural cavity of each mouse was washed with 10 ml of cold PBS, and the parasites were isolated and fixed in PBS-4% paraformaldehyde for further analysis.

### Assessment of Filarial Survival, Development, and Fertility

Filarial survival is assessed at the experiment's endpoint (percentage of the number of larvae recovered during necropsy / number of larvae inoculated at D0). Parasite survival does not differ significantly between any of the strains used until D40 p.i. given the numbers of mice used (see supporting figures and our previous publications [31]). To evaluate parasite development, *L. sigmodontis* larvae were assessed individually at the endpoint of each experiment with a camera lucida-mounted microscope. The parasites' developmental stage and the progress of their moulting between D7 and D12 p.i. were assessed with the morphology of their buccal capsule and by the presence of all, part, or none of the L3 cuticle overlaying the L4 cuticle [65]. Because worm length correlates well with worm stage, we also compared larval length as a more discrete, and thus more sensitive, indicator of developmental rate. To assess filarial sexual maturation, onset of reproduction, and fertility, microfilariae were counted in 10 µl of peripheral blood onwards from D50 p.i., daily for 2 wk, then twice a week until D120 p.i., in four separate experiments of 5–6 mice per group. The blood was immediately mixed with 400 µl of lysis buffer (BD, Cat. # 349202) and stored at room temperature. Counting was carried out after spinning each sample for 3 min at 3000×g, re-suspending the pellet in 40 µl of the same buffer, and then spreading the entire suspension on a microscope slide. All the microfilariae in each sample were counted on an inverted microscope at a magnification of ×50. For analysis, microfilariae counts were averaged per mouse over 10-d windows to reduce day-to-day variations.

### Eosinophil Depletion

Eosinophils were depleted from BALB/c mice by a single injection of 1 mg monoclonal rat anti-CCR3 6S2-19-4 [50] intraperitoneally, 24 h before infection with *L. sigmodontis*. 1 mg rat IgG of the same isotype was used as a control. Efficacy of the depletion was assessed by eosinophil enumeration on cytospins.

### Leukocyte Extraction and Differential Identification

The effect of rIL-5 on subcutaneous eosinophil recruitment was assessed as follows. rIL-5 or BSA were mixed with DMSO 1∶1 and applied on the ears of BALB/c mice. After 4 h, the mice were sacrificed and their ears taken and briefly immersed in 70% ethanol. They were left to dry for 5 min, and the two faces were pulled apart and set to float face down atop 1 ml RPMIc (RPMI 1640, 10% FCS, 100 U penicillin, 100 µg streptomycin, 2 mM glutamine) in 24 well plates overnight at 37°C, 5% CO2. The adherent cells were detached with PBS - 3 mM EDTA - 10 mM glucose, and all cells were harvested and span onto cytospins.

Subcutaneous leukocytes, pleural exudate cells, and tail blood smears were stained with Diff-Quick (Reagena, Finland) and the relative proportions of eosinophils, neutrophils, macrophages/monocytes, and lymphocytes estimated from at least 300 cells per sample.

### Statistical Analysis

The choice of statistical tests was based on sample sizes and on the F test for homogeneity of variances when normal distributions of the errors were expected. Microfilarial count data followed a negative binomial distribution, and homoscedasticity was assessed with the Fligner-Killeen test of homogeneity of variances. In the latter case, data from separate experiments were pooled when possible. Student's unpaired two-tailed *t* test, Chi^2^ or Fisher's Exact test, the Wilcoxon rank-sum test, ANOVA, or Kruskall-Wallis's H-test were used to compare filarial lengths, moulting, and recovery rates depending on sample sizes, normality, and homoscedasticity of the errors. When samples allowed, ANOVA accounting for nesting of mouse and/or mouse sex within experimental group were used instead of non-parametric tests. Generalized linear models were used to assess the effect of treatment, experiment, mouse strain, and day of sampling on microfilaraemia data. R [66] and GraphPad Prism were used for data analyses and representation.

## Supporting Information

Figure S1 ***Litomosoides sigmodontis*** **life cycle.** The infective larva (L3) infects the definitive host (*Sigmodon hispidus* naturally, *Mus musculus* and *Meriones unguiculatus* in the laboratory) via subcutaneous inoculation by the vector *Ornithonyssus bacoti* during a blood feed, or experimentally by needle inoculation. From an initial cohort of infecting larvae, 60% (in primary infections) to 80% (in vaccinated or repeatedly exposed hosts) die in the skin, on average. The survivors migrate through the lymphatic vasculature and reach the pleural cavity after 4 d, where they remain thereafter. Seven days post inoculation, the larvae begin moulting to the 4th larval stage (L4). After 3 more weeks, the L4 moult and become adults. Microfilariae are only detected after D50 in the peripheral blood of the hosts. Our present work analyzes the immunological sources of the variability in these life history trait schedules.
(0.79 MB EPS)

Figure S2 **Direct effects of IL-5 on worm survival, systemic eosinophilia, and on in vitro development of infective larvae.** (A) Worm survival was not affected by the lack of IL-5 in genetically deficient C57BL/6 mice (data identical at D10 and D30 p.i.) nor (B) by the ablation of eosinophils in PHIL mice compared to their wild type C57BL/6 controls. (C) Neither rIL-5 nor α-CCR3 treatments altered worm survival in BALB/c mice.
(0.61 MB EPS)

Figure S3 **Absence of adaptive immunity impairs eosinophil recruitment but has no effect on parasite survival.** (A) The enumeration of cell types in the pleural cavity of infected mice at D30 p.i. revealed that the proportion of eosinophils was lower in rag^−/−^il-4^−/−^ than in wild type C57BL/6 mice (** *p* = 0.008, Wilcoxon rank-sum test, *n* = 5 mice, error bars represent s.e.m.). (B) No effect of adaptive immunity on parasite survival was observed between groups, as is expected in primary infections within 30 d p.i.
(0.57 MB EPS)

## Abbreviations

D p.i.: day post infection
IL-5: Interleukin-5
rIL-5: recombinant IL-5
Th2: T helper 2
